# Editorial: Advances in discoveries of plant phytochemicals

**DOI:** 10.3389/fpls.2024.1414150

**Published:** 2024-04-30

**Authors:** Rajesh Chandra Misra, Ramesha Thimmappa, Mercedes Bonfill

**Affiliations:** ^1^ Biochemistry and Metabolism Department, John Innes Centre, Norwich, United Kingdom; ^2^ Amity Institute of Genome Engineering, Amity University, Noida, Uttar Pradesh, India; ^3^ Department of Biology, Healthcare and Environment, Faculty of Pharmacy and Food Sciences, University of Barcelona, Barcelona, Spain

**Keywords:** phytochemicals, bioactivity, metabolism, biosynthetic pathways, metabolic engineering, heterologous expression

In the realm of health and medicine, the pursuit of novel therapeutic agents often leads scientists to explore the hidden riches of nature. Among these treasures, plant phytochemicals stand out as a diverse and promising category of compounds with profound implications for human health. Recent advances in the discovery and understanding of these phytochemicals have unveiled a wealth of opportunities that could revolutionize medicine, nutrition, and even environmental as well as agricultural sustainability. Phytochemicals are naturally occurring compounds found in plants, encompassing a wide array of chemical classes such as flavonoids, alkaloids, terpenes, and phenolic compounds. While plants produce phytochemicals primarily for their own defence against environmental stressors, these compounds also exhibit remarkable bioactivity when consumed by humans. From antioxidant and anti-inflammatory properties to potential anticancer effects, the therapeutic potential of phytochemicals has captivated researchers worldwide. The therapeutic potential of plant phytochemicals extends across a wide range of health conditions. For instance, flavonoids, abundant in fruits and vegetables, have been linked to cardiovascular health, cognitive function, and even longevity (Panche et al., 2016). Meanwhile, polyphenols found in tea, cocoa, and red wine have garnered attention for their antioxidant properties and potential role in preventing chronic diseases such as cancer and neurodegenerative disorders (Pandey and Rizvi, 2009). Despite these remarkable advancements, challenges remain in harnessing the full potential of plant phytochemicals. Issues such as bioavailability, toxicity, and standardization pose hurdles to their development as pharmaceuticals and functional foods (Atanasov et al., 2015). Moreover, biologically active phytochemicals are regulated by a variety of factors including genetics, environmental conditions, and developmental stages of the plant that involve highly complex and sophisticated biosynthetic pathways (Misra et al., 2014, 2015; Verma and Shukla, 2015; Isah, 2019). However, advancement in next generation sequencing (NGS) and bioinformatic tools have made the rich metabolism of plants more accessible. Historically, plant pathway elucidation has been a challenge. A comprehensive knowledge of the biosynthetic pathways that generate these high value molecules will assist in their exploitation for a wider variety of applications. For instance, identification of missing enzyme sets for vinblastine biosynthesis, a potent anticancer drug from *Catharanthus roseus* and elucidation of the complete biosynthetic pathway of QS-21, a promising vaccine adjuvant derived from *Quillaja saponaria* (Caputi et al., 2018; Reed et al., 2023; Martin et al., 2024). Additionally, one of the most significant advances in phytochemical research has been the development of new analytical techniques. Advanced analytical techniques, such as mass spectrometry and nuclear magnetic resonance spectroscopy, have enabled scientists to identify and characterize phytochemicals with unprecedented precision that led to the discovery of new phytochemicals in plants that were previously thought to be absent.

The Research Topic includes twelve original research articles and three review articles, with a special focus on the new discoveries of plant phytochemicals as well as their bioactivities. Indeed, plant phytochemicals have garnered considerable attention for their potential therapeutic benefits across various health conditions. For example, Kim et al., investigates the impact of salicylic acid (SA) treatment on secondary metabolites in soybean roots and their potential anti-LDL (low-density lipoprotein) oxidation effects. The authors found that SA treatment led to significant changes in secondary metabolites in soybean roots in particular, SA stimulated the production of coumestrol, a beneficial compound, and broke down its precursors (coumestrin and malonylcoumestrin). These alterations included increases in certain compounds known for their antioxidant properties. Moreover, the authors observed that extracts from SA-treated soybean roots exhibited a much stronger ability to prevent LDL cholesterol oxidation compared to untreated roots, suggesting potential health benefits related to cardiovascular health. Further, Qi et al., reports the anti-acetylcholinesterase (anti-AChE) potential and alkaloid composition of *Rhizoma Coptidis* (RC) from different *Coptis* species. It employs a combined approach involving spectrum-effect relationship analysis and molecular docking. The study suggests *Coptis teeta* might be the best source of RC for Alzheimer’s treatment. Extracts from this species showed the strongest inhibitory activity against acetylcholinesterase (AChE), an enzyme involved in Alzheimer’s progression. Three alkaloids, columbine, berberine, and palmatine, were pinpointed as the main contributors to AChE inhibition. These can be used as markers for selecting the best RC source for Alzheimer’s treatment. Furthermore, molecular docking simulations supported the findings, indicating strong binding between these key alkaloids and the active site of AChE, which provides insights into the mechanisms underlying their activity. In another study, Joshi et al., inspected if *Cyamopsis tetragonoloba* (guar) seed extract could enhance the antioxidant activity of existing phenolic phytochemicals. The authors found that guar seed extract, at low concentrations, significantly increased the antioxidant activity of epigallocatechin gallate (EGCG), a powerful antioxidant. This suggests guar seed extract has potential as an antioxidant booster. The extract also showed promise in protecting cells from oxidative stress in lab tests. Finally, the authors identified previously unknown metabolites in guar extract, which might explain its antioxidant-enhancing effect. The findings could contribute to understanding the synergistic effects of plant extracts and phenolic compounds in combating oxidative stress, which is crucial for potential applications in food, pharmaceuticals, or nutraceuticals. These discoveries underscore the importance of a plant-rich diet in promoting overall health and well-being. Additionally, Kyriakou et al., examines the chemical composition of watercress, focusing on polyphenolics, glucosinolates, and isothiocyanates present in its aerial parts. The authors isolated and identified these compounds in the aerial parts of watercress using analytical techniques like liquid chromatography with tandem mass spectrometry and were also able to quantify the amounts of each compound present. Interestingly, they found that the content of isothiocyanates (potentially beneficial compounds) depended on the presence of other glucosinolates, not just individual ones. Overall, this research provides a detailed analysis of the major health-promoting compounds in watercress, paving the way for using different watercress parts for potential future therapies.

Furthermore, the exploration of plant phytochemicals holds promise for addressing pressing global challenges, including human health and environmental pollution. Plant-derived compounds have shown antibacterial, antifungal, and antiviral activities, offering new avenues for combating infectious diseases in an era of dwindling antibiotic efficacy (Orhan et al., 2010; Polturak et al., 2023). Additionally, phytochemicals can play a role in sustainable agriculture by serving as alternatives to synthetic pesticides and fertilizers, thus reducing the ecological footprint of farming practices. A study by Liu et al., identified four sesquiterpenes from *A. artemisiifolia* and examined their impact on neighbouring plants. They found that these sesquiterpenes exhibited allelopathic effects, affecting the germination and growth of other plants. This research sheds light on the potential role of sesquiterpenes in the ecology and competitive interactions of *A. artemisiifolia*. In a study by Alruhaili et al., revealed the phytochemical composition and antimicrobial properties of two plants, *Amomum subulatum* and *Amomum xanthioides*, through both experimental and computational approaches. The authors analysed the chemical constituents of these plants including protein, lipids, and essential oils and evaluated their antimicrobial activity against various pathogens. Interestingly, *A. subulatum* had higher levels of carvacrol, a compound known for its antimicrobial properties, compared to *A. xanthioides*. Extracts from both plants showed antioxidant activity, with *A. subulatum* seeds having the strongest effect. Additionally, the study revealed that *A. subulatum* extracts were particularly effective against several harmful bacteria species, which could be explored for therapeutic purposes. Further, the *in silico* analysis provides insights into the mechanisms through which these phytochemicals may exert their antimicrobial effects. A systematic elucidation of natural product biosynthetic pathways, leading to a better understanding of how these valuable compounds are made and provide opportunities for metabolic pathway engineering. For example, Almeida et al., established hairy roots of *Cucurbita pepo* as a platform to modify and increase production of cucurbitacins, which are valuable plant compounds with potential medicinal applications. The authors aim to increase the yield of cucurbitacins by manipulating the metabolic pathways in *Cucurbita pepo* through genetic engineering techniques. They showed that overexpression of *CpCUCbH1* (bHLH transcription factor) can induce cucurbitacins in several Cucurbitaceae species, and also overexpression of the cytochromes P450 *CsCYP88L2* and *McCYP88L7* from *Cucumis sativus* and *Momordica charantia* (respectively), results in accumulation of new analogues of cucurbitacin with distinct structural modifications that are previously unknown. The study provides initial evidence that a hairy roots platform can be used in modifying and increasing the production of valuable plant specialized metabolites for which the biosynthetic pathway has not been fully characterized. Furthermore, Istiandari et al., investigates the roles of Class I and Class II NADPH cytochrome P450 reductases in triterpenoid biosynthesis within *Lotus japonicus*. The authors found that these two classes of reductases play distinct roles in the biosynthesis process. Class I CPR, encoded by the *LjCPR1* gene, seems to be crucial for plant growth and development, particularly seed development. Class II CPRs, encoded by *LjCPR2* genes, are more involved in the specific production of soyasaponins, a type of triterpenoids. The findings suggest that this difference arises because Class I CPRs are generally involved in basic plant metabolism, while Class II CPRs are specialized for synthesizing particular compounds like soyasaponins. This finding contributes to our understanding of how plants produce complex molecules through the interplay of different enzymes. Finally, Zhang et al., reviewed how advancements in high-throughput omics technologies are revolutionizing the discovery of new drugs from medicinal plants. Omics technologies encompass genomics, transcriptomics, proteomics, and metabolomics, which allow for comprehensive analysis of biological systems. By applying these technologies to medicinal plants, researchers can identify and characterize bioactive compounds with potential therapeutic applications. This integrated approach offers a more efficient and systematic way to discover novel drugs from natural sources compared to traditional methods. The review highlights the benefits and challenges of employing omics technologies in medicinal plants research and underscores their significant role in advancing natural drug discovery.

In conclusion, the recent strides in the discovery and characterization of plant phytochemicals represent a watershed moment in pharmaceuticals. These natural compounds offer a treasure trove of therapeutic opportunities, from disease prevention to environmental stewardship. As scientists continue to unravel the mysteries of the plant kingdom, we can look forward to a future where the healing power of nature is fully realized for the benefit of humanity. It is imperative that we support and invest in further research endeavours to unlock the full potential of these botanical wonders.

## Challenges and future prospects

The field of phytochemical research has seen significant advances in recent years, with ongoing discoveries of new plant compounds and a growing understanding of their potential health benefits. Despite the many advances in phytochemical research, there are still some challenges that need to be addressed. One challenge is the lack of standardization in the production of phytochemical extracts. This can make it difficult to compare the results of different studies and to develop reliable and effective phytochemical-based products. Another challenge is the need for more clinical trials to evaluate the safety and efficacy of phytochemicals. Although many phytochemicals have shown promise in preclinical studies, more research is needed to confirm their benefits in humans. However, the advances in the discovery and understanding of plant phytochemicals have the potential to revolutionize the way we approach nutrition and preventive healthcare. Researchers are continuing to make new discoveries about the health benefits of phytochemicals and to develop new and innovative ways to use them to prevent and treat disease. One promising area of research is the development of personalized phytochemical-based therapies. This could lead to more effective and targeted treatments for a variety of diseases. Another promising area of research is the combination therapy where phytochemicals can be used in combination with conventional drugs to improve efficacy and reduce side effects.

## Author contributions

RCM: Conceptualization, Resources, Visualization, Writing – original draft, Writing – review & editing. RT: Writing – original draft, Writing – review & editing, Funding acquisition. MB: Writing – original draft, Writing – review & editing.
